# NeuroCypher ASD: Machine Learning with Knowledge Graph-Based Representation for ASD Screening in Toddlers

**DOI:** 10.1007/s41666-026-00243-x

**Published:** 2026-06-23

**Authors:** Georgios Bouchouras, Dimitrios Doumanas, Konstantinos Kotis

**Affiliations:** 1 Intelligent Systems Lab, Department of Cultural Technology and Communication, University of the Aegean, University Hill, Mytilene, 81100 Lesvos Greece; 2 Department of Sports Coaching and Physical Education, School of Sports Sciences and Physical Education, Metropolitan College, 46 Egnatia Str, Thessaloniki, 54625 Central Macedonia Greece

**Keywords:** Autism spectrum disorder, Knowledge graphs, Natural language interfaces, Explainable artificial intelligence, Early childhood screening

## Abstract

Autism Spectrum Disorder (ASD) screening often relies on structured questionnaires. Yet these data are not always easy to interpret or use in practice. There is a need for screening frameworks that support risk estimation, case comparison, and pattern exploration in a clear and accessible way. In this work, we present NeuroCypher ASD, a framework that combines tabular machine learning with a knowledge graph for ASD screening, interpretation, and data exploration. The tabular model uses questionnaire responses and demographic features to estimate ASD risk. The knowledge graph organizes the data and supports case comparison, pattern exploration, and natural language querying. The system is evaluated under a leakage-aware protocol using tabular, graph-based, and hybrid models. The results show strong performance for the tabular model and clarify the role of graph-based representations within the overall workflow. In this setting, the knowledge graph supports structured analysis of screening data by helping users examine relationships between cases, inspect shared features, and interpret predictions in context. To strengthen interpretability, the framework includes SHAP-based analysis and approximate mappings between embedding dimensions and original questionnaire features. It also includes an anomaly detection module that highlights atypical cases for closer review. Overall, NeuroCypher ASD provides a practical framework for combining prediction, interpretation, and data exploration in ASD screening. The results show the value of integrating machine learning, graph-based context, and natural language interaction in more transparent and accessible screening workflows, particularly in school-based environments.

## Introduction

Autism Spectrum Disorder (ASD) is a neurodevelopmental condition associated with difficulties in social interaction and communication, along with repetitive behaviors or restricted interests [10, 15, 19]. Over the past two decades, ASD diagnoses have increased worldwide, increasing the need for accurate, timely, and scalable screening approaches [24–26]. Early identification, especially during the toddler years, can improve developmental outcomes by enabling earlier support and more targeted intervention [14].

Despite the availability of screening tools such as the Q-Chat, translating questionnaire responses into meaningful insights remains challenging in practice. In real-world settings, professionals often work with heterogeneous datasets and large numbers of cases, while available tools typically require technical expertise that is not always accessible in real-world settings [2–4]. As a result, structured screening data is often underutilized in everyday decision-making processes.

This challenge is particularly evident in school environments, where occupational therapists and school psychologists are often among the first to notice behavioral concerns. In these settings, there is a need for systems that not only provide risk estimates but also support interpretation, comparison across cases, and exploration of behavioral patterns. Tools that allow users to interact with screening data, identify similarities between cases, and relate individual profiles to broader patterns can support earlier recognition of concerns and more informed referral decisions.

Knowledge graphs (KGs) provide a useful way to organize complex health-related information by representing data as connected entities and relationships [20]. In ASD screening, this makes it possible to connect questionnaire responses, demographic information, family history, and outcomes within a single structure that supports exploration and interpretation [16]. However, the practical use of KGs remains limited because graph-based systems often depend on specialized query languages and interfaces that are difficult for non-technical users to adopt [35].

In this work, we present NeuroCypher ASD, a framework that combines tabular machine learning with a knowledge graph for ASD screening. The model uses questionnaire responses and demographic features to estimate ASD risk. This process follows the structure of the screening data, while the knowledge graph adds context. It supports case comparison, pattern exploration, and interpretation of results.

To better understand the role of each component, the framework explicitly evaluates multiple predictive settings, including tabular-only, graph-based, and hybrid models. This design allows the contribution of graph-derived representations to be examined alongside standard tabular approaches, rather than assumed a priori.

The framework is built around a Neo4j-based knowledge graph that integrates questionnaire responses, patient metadata, and ASD-related outcomes into a structured representation. A central feature of the system is a natural-language interface that allows users, including clinicians, occupational therapists, and educators, to interact with this graph without requiring technical query skills. By using a large language model (LLM) to translate user input into Cypher queries, the framework enables direct access to the underlying data and supports real-time exploration of behavioral and demographic patterns in a form that is easier to interpret and use in practice.

In addition to prediction and exploration, the framework includes an anomaly detection component that highlights cases with unusual profiles relative to the dataset. This mechanism supports decision-making by identifying atypical or uncertain cases that may require closer inspection.

Overall, the proposed framework is designed around a separation of roles between predictive modeling and graph-based contextual analysis. The tabular model provides an estimate of ASD risk, while the knowledge graph allows users to examine how this estimate relates to behavioral patterns, demographic context, and similar cases within the dataset. In this setting, context-aware analysis refers to the interpretation of individual cases in relation to these interconnected elements, rather than in isolation.

This study is guided by the question of how machine learning, knowledge graphs, and natural-language interfaces can be combined to make ASD screening data more accessible, interpretable, and useful in school-based settings.

The work makes three main contributions. First, it presents a structured framework that combines tabular prediction with graph-based organization and exploration of screening data. Second, it shows how natural-language interaction can make structured ASD screening data more accessible to non-technical users. Third, it provides a practical workflow that supports screening, interpretation, and case review within a single system.

The remainder of this paper is organized as follows. Section 2 reviews related work in ASD screening and semantic technologies. Section 3 describes the proposed methodology, including data processing, knowledge graph construction, and machine learning components. Section 4 presents the experimental results, and Section 5 discusses the findings, concludes the paper, and outlines directions for future research.

## Related Work

### Artificial Intelligence (AI)/Machine Learning (ML) in ASD Diagnosis and Prediction

Wankhede et al. [32] explore the integration of artificial intelligence (AI) in the diagnosis and treatment of autism spectrum disorder, focusing on AI’s capabilities in behavioral analysis, neuroimaging, genetic profiling, and therapeutic interventions such as virtual reality and robot-assisted systems. Their review highlights how machine learning algorithms enhance early detection and personalize treatment by analyzing multimodal data including speech, facial cues, and clinical records. They also discuss AI-driven tools for augmentative communication and social skills training. While the paper presents a comprehensive overview, it is largely conceptual and lacks empirical validation through clinical trials, limiting its translational applicability.

Moridian et al. [17] examine the application of AI techniques for the automatic detection of autism spectrum disorder using structural and functional MRI modalities. Their comprehensive review examines both machine learning and deep learning approaches in CAD systems, detailing preprocessing techniques, feature extraction, and classification methods. The study also compares the performance of various algorithms and highlights the importance of public neuroimaging datasets like ABIDE. Despite its thorough scope, the work largely synthesizes existing literature and lacks a unifying framework or evaluation of clinical deployment, limiting its practical contribution to diagnostic implementation.

Sundas et al. [29] analyze the role of AI and Internet of Things (IoT) technologies in the diagnosis and management of autism spectrum disorder, with a focus on wearable sensors, smart environments, and intelligent devices. Their systematic literature review evaluates 28 studies and introduces a taxonomy categorizing approaches into diagnostic tools and quality-of-life enhancers for autistic children. The study analyzes technical aspects like data mining, deep learning, and sensor integration, offering insights into performance metrics such as accuracy and sensitivity. However, the review is limited by a lack of empirical validation and real-world deployment, reducing its immediate clinical applicability.

Haque et al. [9] evaluate the performance of classical machine learning algorithms—Logistic Regression, Support Vector Classifier, K-Nearest Neighbour, Decision Tree, and Random Forest—for early autism spectrum disorder detection in toddlers and children. Using publicly available and real-world datasets from Bangladesh, they demonstrate exceptionally high accuracy and mIoU scores, particularly with SVM and Logistic Regression models. Their methodology emphasizes simplicity, interpretability, and computational efficiency, making it suitable for resource-constrained environments. However, the study is constrained by limited dataset diversity and its exclusive focus on structured data, which may reduce generalizability to broader, unstructured real-world scenarios.

### Graph Learning/Knowledge Graphs for ASD

Yousefian et al. [36] investigate the detection of autism spectrum disorder using graph representation learning algorithms applied to functional MRI data from the ABIDE I and II datasets. They evaluate several shallow and feature-based embedding techniques—such as Node2vec, AWE, and Graph2Img—combined with a CNN classifier to identify connectivity patterns distinguishing ASD from controls. Their results demonstrate improved performance in leave-one-site-out validation, particularly with Graph2Img, achieving up to 80% accuracy. The study highlights the benefits of embedding-based dimensionality reduction for cross-site generalizability. However, the approach lacks neurobiological interpretability and fails to localize the altered brain regions, limiting its clinical transparency.

Yang et al. [34] explore the diagnosis of autism spectrum disorder using a novel approach that integrates Pearson’s correlation with spatial constraints to construct functional brain networks from resting-state fMRI data. Their method, termed Pearson’s correlation-based Spatial Constraints Representation (PSCR), enhances the estimation of brain connectivity by incorporating anatomical proximity, which is then fed into a graph attention network (GAT) for classification. The study demonstrates that PSCR combined with GAT achieves superior accuracy and robustness compared to other graph-based and machine learning frameworks. However, the method is limited by its reliance on a single imaging modality and does not account for group-level variability or multimodal integration.

Wei et al. [33] present a graph neural network-based framework for autism spectrum disorder diagnosis by integrating diffusion tensor imaging (DTI) and resting-state fMRI data. Their method constructs brain connectivity graphs using DTI as the adjacency matrix and fMRI as node features, enabling bimodal representation learning. They introduce a regularization term that optimizes inter-class separation and intra-class compactness using Wasserstein graph distance, which enhances classification performance. The model also performs eigenvalue-based analysis to identify brain regions potentially associated with ASD. While the approach achieves high diagnostic accuracy, it lacks validation on larger or more diverse datasets, limiting generalizability.

### LLMs, NLP, and ASD-Driven Dialogue Systems

Garg et al. [7] examine the potential of LLMs, specifically ChatGPT-4, in enhancing the early prediction of autism spectrum disorder by leveraging its advanced natural language processing capabilities. They highlight GPT- 4’s strengths in recognizing subtle linguistic patterns, integrating diverse data sources, and supporting scalable, accessible screening tools. The study also considers GPT-4’s interdisciplinary flexibility in processing psychological, medical, and educational information. Despite these advantages, the authors caution against limitations such as data bias, ethical concerns, and over-reliance on AI in clinical settings. Their work remains conceptual and lacks empirical evaluation, constraining its immediate clinical relevance.

Papadopoulos et al. [23] assess the implications of LLMs, such as ChatGPT, for autistic and neurodivergent individuals, emphasizing both the supportive potential and associated risks. They highlight benefits including simplified information access, non-demanding communication environments, and tools for learning and social interaction. The commentary also critiques LLMs for perpetuating medicalized views of autism, spreading misinformation, and inadequately representing non-speaking individuals. A key contribution is the call for neurodiversity-affirming development practices and collaborative AI design. However, the study is conceptual and anecdotal, lacking empirical data or formal evaluation, which limits its scientific generalizability.

Yeung et al. [5] describe the development and deployment of a clinical natural language processing (NLP) service within the UK’s National Health Service to extract structured insights from unstructured electronic health records. Their approach leverages domain-expert annotations using the MedCAT toolkit for named entity recognition and linking, enabling downstream applications such as clinical coding, disease tracking, and patient timeline generation. They outline a robust implementation framework and propose future extensions involving fine-tuning LLMs grounded in real-world clinical knowledge. However, the study is limited by a lack of formal performance benchmarks across diverse healthcare settings.

Chu et al. [6] introduce ChatASD, a dialogue framework that enhances LLMs with autism-specific KGs to improve the accuracy of autism-related question-and-answer systems. The system integrates Retrieval-Augmented Generation (RAG) with structured medical knowledge sourced from curated literature, guidelines, and clinical expertise. Their method leverages community-based entity clustering and GPT-4o-powered knowledge extraction to deliver domain-accurate and context-rich responses. Evaluated on custom benchmarks (AQA-R and AQA-P), ChatASD outperforms general LLMs in accuracy, relevance, and fidelity. While promising, the framework’s reliance on manual data curation and its limited validation outside experimental settings pose constraints for real-world scalability.

Niu et al. [21] design an intelligent autism knowledge learning and service interaction platform that integrates multiple stakeholders—patients, families, educators, and institutions—into a dynamic, user-centered ecosystem. The platform combines heterogeneous data sources, personalized recommendation algorithms, and domain-specific KGs to support autism education, consultation, and awareness. Key components include user modeling, resource recommendation, and multi-source knowledge extraction using advanced techniques like decision trees and graph neural networks. Although the system demonstrates a promising framework, its current prototype lacks comprehensive validation, and real-world deployment has been limited to small-scale volunteer testing.

### Summary

Despite notable advances in the application of AI and machine learning to ASD, existing approaches present several critical limitations. Many studies focus on isolated modalities, such as neuroimaging or behavioral data, without offering integrated frameworks for clinical decision-making. For example, Yousefian et al. [36] and Yang et al. [34] rely exclusively on fMRI data, which limits interpretability and accessibility in real-world clinical settings. Others, like Wankhede et al. [32] and Moridian et al. [17], provide comprehensive reviews but lack empirical validation or system deployment, reducing their translational value. While Garg et al. [7] and Chu et al. [6] explore the promise of LLMs and KGs, their frameworks depend heavily on manual curation or remain at the proof-of-concept level. Additionally, tools described by Niu et al. [22] and Haque et al. [9] either lack real-time interaction capabilities or are restricted to structured datasets, which limits flexibility and user accessibility for non-technical practitioners.

Overall, existing work in ASD screening tends to focus either on predictive performance or on isolated data modalities, often without addressing how results can be interpreted and used in practice. In particular, there is a gap between questionnaire-based screening models, which can achieve high accuracy, and systems that allow users to explore, interpret, and contextualize these results in a structured and accessible way.

The proposed NeuroCypher ASD framework addresses this gap by combining tabular prediction with graph-based data organization and natural-language interaction within a single workflow. The system uses questionnaire and demographic data to estimate ASD risk, while the knowledge graph enables structured exploration of cases, identification of similar profiles, and inspection of behavioral and demographic patterns. By allowing users to query the data using natural language and interact with it through a graph representation, the framework supports interpretation and exploration beyond prediction alone.

## The NeuroCypher ASD Framework

The NeuroCypher ASD Framework is a modular and interpretable system designed to support school-based autism spectrum disorder (ASD) screening through structured data exploration rather than standalone automated diagnosis. The framework integrates a Neo4j knowledge graph, graph representation learning, machine learning, anomaly detection, and a natural-language querying interface. Its main purpose is to help clinicians, educators, and therapists inspect behavioral profiles, compare cases, and identify patterns in screening data through a transparent and queryable representation. Furthermore, to ensure full research transparency and facilitate methodological reproducibility, the complete analytical codebase and the end-to-end computational pipeline have been made accessible in a public repository, as detailed in the Declarations section.

The workflow begins with ingestion of questionnaire and demographic data from the Q-Chat-10 toddler screening dataset. After preprocessing, each case is represented as a :Case node connected to behavioral, demographic, and submitter-context nodes. The resulting graph supports both direct Cypher querying and embedding-based downstream analysis. To enable relational representation learning, Node2Vec embeddings are generated from the graph and used as graph-level features in supervised experiments. In parallel, an Isolation Forest module is used for anomaly detection to flag atypical or potentially ambiguous cases. A large language model is integrated as a natural-language-to-Cypher layer, enabling non-technical users to query the graph without knowledge of graph query syntax.

**Fig. 1:**
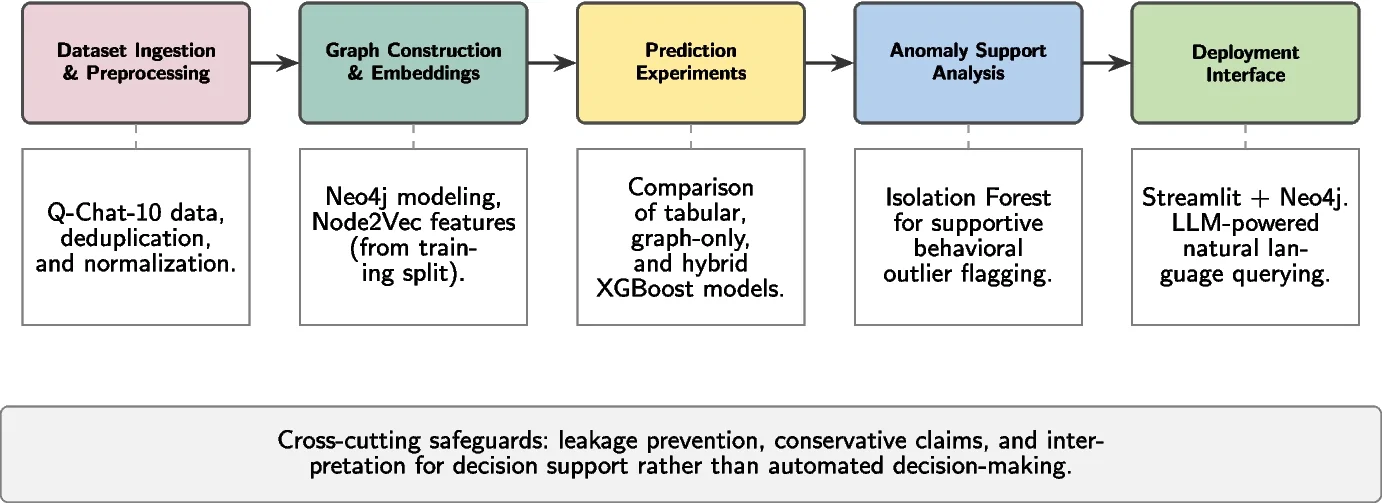
Overview of the NeuroCypher ASD framework. Full implementation details and code availability are provided in the Declarations section

Figure 1 summarizes the main stages of the framework, from data ingestion and graph construction to predictive evaluation, anomaly flagging, and user interaction. The design separates prediction from contextual interpretation: tabular modeling is used to estimate screening risk, whereas the graph and query interface are used to inspect how cases relate to one another and how patterns appear across the dataset.

### Dataset Description and Preprocessing

This study used the *Autistic Spectrum Disorder Screening Data for Toddlers* [30, 31], comprising 1,054 samples and questionnaire- and demographic-level variables (see Appendix A). The dataset includes ten Q-Chat behavioral items (A1–A10), age in months, demographic characteristics, and the identity of the respondent. The provided target variable, Class_ASD_Traits, is generated by the screening logic of the source application. To avoid redundancy and potential information leakage, Qchat-10-Score was excluded from modeling, as it is directly computed from the questionnaire responses.

Preprocessing was performed with pandas. Numeric fields were parsed using locale-aware conversion, text fields were stripped of whitespace and normalized, and cases with missing identifiers or labels were removed. To reduce optimistic bias, duplicate rows were removed before model splitting. This step removed 79 duplicated instances from the original dataset, yielding 975 unique rows for evaluation.

All preprocessing decisions were completed before supervised evaluation. After cleaning and deduplication, the dataset was partitioned using a deterministic stratified split in order to preserve class balance while preventing overlap between training and holdout cases. This ordering was important because the graph, embeddings, and downstream models were all derived from the post-cleaning split rather than from the full dataset.

### Knowledge Graph Construction and Embedding Generation

Each preprocessed case was inserted into Neo4j as a :Case node. Behavioral answers were connected to :BehaviorQuestion nodes through HAS_ANSWER relationships, demographic values were connected through HAS_DEMOGRAPHIC, and respondent information through SUBMITTED_BY. Additional graph variants included optional behavior-pattern, complexity, similarity, and demographic-context edges for ablation and sensitivity analysis.

The graph schema was designed to preserve both individual case information and relations shared across cases. In this structure, a case can be explored directly through its questionnaire responses and metadata, or indirectly through its connections to broader behavioral and demographic patterns. This allows the graph to function both as a data store for querying and as the basis for graph representation learning.

**Fig. 2:**
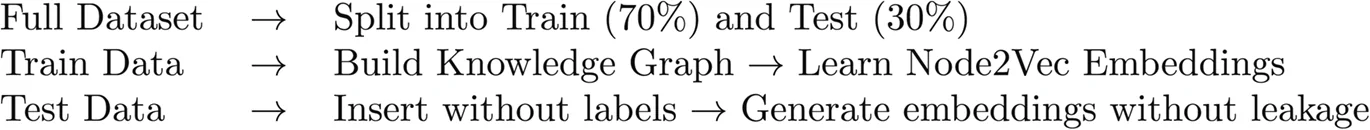
Graph embedding generation workflow. Embeddings are learned from the training data, while test cases are inserted without labels to avoid information leakage

Graph embeddings were generated with Node2Vec in the Python pipeline rather than through in-database graph analytics. The current implementation writes 128-dimensional embeddings back to the case nodes and uses a fixed random seed of 42 for reproducibility. To reduce transductive contamination, the protocol first split the deduplicated dataset into a 70/30 stratified train/test partition. The graph was then built only from the training cases. Test cases were inserted as unlabeled nodes for embedding refresh without exposing diagnostic labels during graph generation. This design does not eliminate all limitations of the underlying screening task, but it substantially reduces leakage relative to the original all-cases graph construction (Fig. 2).

Node2Vec was selected because it provides a simple and reproducible way to convert graph structure into dense vector representations suitable for downstream classification. These embeddings were used as graph-derived features at the case level. In the graph-only setting, they were used as the sole predictive input; in the hybrid setting, they were concatenated with the raw tabular variables.

### Graph Variants and Edge Weight Design

To examine how graph construction influenced downstream behavior, we evaluated several graph variants derived from the same base schema. These included a full graph, a simplified graph, a version without similarity edges, a version without demographic-context edges, and an anomaly-gated variant used to test whether excluding atypical profiles changed performance. The purpose of these variants was not to define separate systems, but to clarify which structural components contributed most to relational organization and downstream separability.

In addition to graph topology, we examined whether edge weighting choices affected the learned representations. A baseline heuristic weighting scheme was assigned to the main relationship types in order to reflect their intended structural contribution within the graph. This baseline was then compared with a uniform-weight configuration and with perturbed versions of the heuristic scheme. Perturbation was applied as a controlled global modification of the baseline weights to test whether the graph-based pipeline remained stable under reasonable weighting changes. This analysis was included to assess sensitivity rather than to exhaustively optimize weights.

**Fig. 3:**
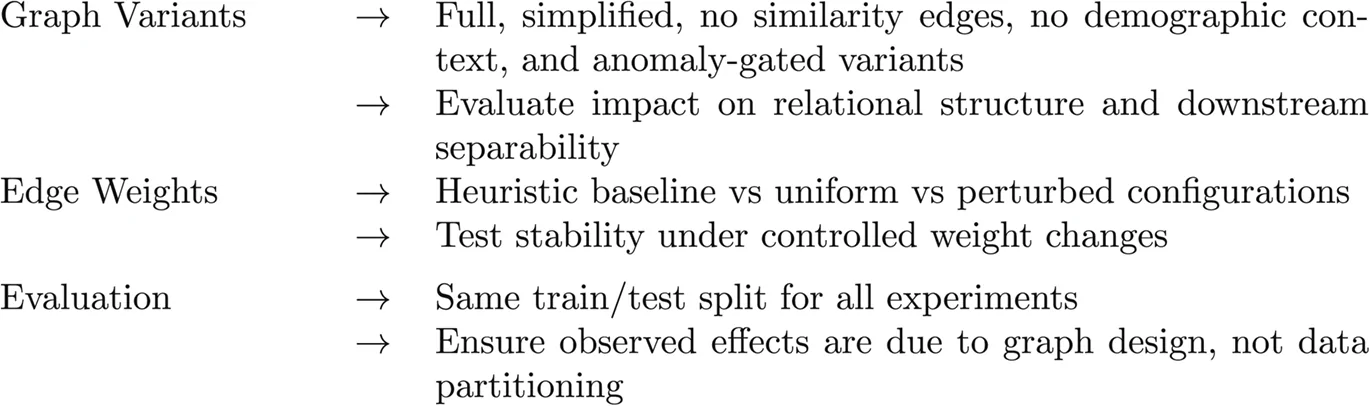
Graph design and sensitivity analysis workflow. Multiple graph variants and weighting schemes were evaluated under a fixed train/test split to assess how structural design choices influence representation and downstream behavior

Because edge weights can influence random-walk-based embedding methods indirectly through graph connectivity, all weighting experiments were conducted under the same train/test partition used in the main predictive evaluation. This ensured that changes in performance could be attributed to graph design choices rather than to differences in case assignment across splits (Fig. 3).

### Predictive Evaluation Design

Three predictive settings were evaluated. First, a *reference tabular baseline* used XGBoost on raw questionnaire and demographic variables (A1–A10, age in months, sex, ethnicity, jaundice history, family ASD history, and respondent type). Second, a *graph-only baseline* used XGBoost on Node2Vec embeddings derived from the training graph. Third, a *hybrid model* combined raw tabular variables with graph embeddings. All experiments used a deterministic 70/30 stratified split with 5-fold stratified cross-validation on the training partition only. To account for class imbalance in the ASD trait label, the training procedure used cost-sensitive learning rather than synthetic oversampling: Logistic Regression and Random Forest used class weighting, while XGBoost used scale_pos_weight computed from the training partition. Hyperparameters for XGBoost were selected through a small CV-guided search over depth, learning rate, number of trees, subsampling, column sampling, and regularization settings.

Evaluation metrics included ROC AUC, F1-score, precision, recall, and average precision. These metrics were computed on the untouched holdout set after all preprocessing, embedding generation, and parameter selection decisions were completed on the training partition. For clarity, ROC AUC measures how well the model separates positive from negative cases across decision thresholds, with higher values indicating better overall discrimination. CV ROC AUC denotes the mean ROC AUC across the cross-validation folds of the training set, whereas holdout ROC AUC denotes performance on the independent test set. The former reflects model behavior during development, while the latter reflects generalization to unseen data. Precision measures how often cases predicted as positive are actually positive, whereas recall measures how many of the true positive cases are successfully identified. The F1-score summarizes the balance between precision and recall. Average precision (AP) summarizes performance across the precision–recall curve and is particularly useful when class balance is uneven. The term holdout refers to the independent test partition that was not used during model fitting or parameter selection. This evaluation design was chosen to separate three roles within the framework: direct prediction from tabular screening inputs, prediction from graph-derived structure, and prediction from their combination. The comparison was therefore used to understand the contribution of each representation under the same evaluation protocol, rather than to assume that graph-derived features would necessarily outperform standard tabular inputs.

**Fig. 4:**
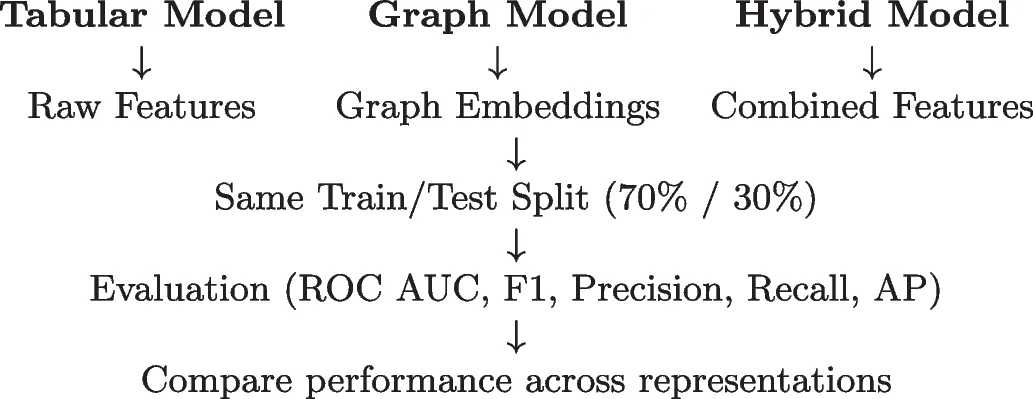
Predictive evaluation pipeline. Three modeling approaches were evaluated under the same data split to compare the contribution of tabular features, graph structure, and their combination

In addition to the main predictive comparison, the anomaly-gated setting was examined as a decision-support variant in which cases flagged as atypical could be treated as requiring closer review. This allowed us to test whether selective handling of unusual profiles altered the behavior of the graph-based pipeline on the holdout data, as shown in Fig. 4.

### Interpretability and Representation Analysis

To improve the interpretability of the graph-based predictive pipeline, we examined which learned embedding dimensions contributed most strongly to model output. This analysis was performed on the embedding-based XGBoost classifier using SHAP values at both the global and case level. Global analysis was used to identify the most influential embedding dimensions across the holdout predictions, whereas local analysis was used to inspect how these dimensions contributed to individual cases.

Because graph embeddings do not correspond directly to named questionnaire items or demographic fields, an additional approximation step was used to relate influential embedding dimensions back to the original variables. This was done by examining the statistical association between selected embedding dimensions and the source features within the training data. The resulting links were used only as interpretive aids to support understanding of what the embeddings may capture; they were not treated as exact semantic mappings.

This interpretability analysis was included to complement predictive evaluation. Rather than treating the graph embeddings as opaque features, the goal was to provide a clearer view of how structural signals in the graph may align with recurring behavioral or demographic patterns in the data.

### Anomaly Detection and Decision-Support Layer

The framework includes an unsupervised anomaly detection component based on Isolation Forest. This module operates on case-level representations in order to flag profiles that differ from the broader dataset pattern. The aim is not to assign diagnostic status, but to identify cases that may benefit from closer review because they appear less typical relative to the remaining sample.

Within the broader workflow, anomaly detection serves as a decision-support layer around the predictive and graph-based components. A case can therefore be examined not only in terms of its predicted label, but also in terms of whether it appears unusual compared with the rest of the dataset. This function is especially useful when cases do not fit common behavioral combinations or when a user wishes to identify profiles that merit additional attention.

### Natural-Language Querying Interface

To support practical use by non-technical users, the graph was coupled with a natural-language interface that translates user questions into Cypher queries. This interface was implemented as an LLM-assisted layer between the user and the Neo4j graph using the OpenAI API, with gpt-4o-mini as the default configured model in the current implementation. Users can therefore request case comparisons, inspect shared questionnaire responses, or explore demographic patterns without directly writing graph queries.

The purpose of this layer is to reduce the technical barrier to using graph-structured data in school-based settings. In methodological terms, it functions as an access mechanism rather than as a predictive component. It does not alter the graph itself or the downstream classifiers, but makes the information stored in the graph easier to retrieve and interpret in real time.

### Data Integrity and Clinical Interpretation

The dataset label is derived from the questionnaire responses. Consequently, very high predictive performance may largely reflect the underlying screening logic rather than independent diagnostic capability.

Accordingly, NeuroCypher ASD is not intended to replace established screening tools through improved accuracy. Instead, it supports structured exploration of behavioral and demographic patterns, enabling case comparison, anomaly flagging, and natural-language interaction with the data.

Consistent with this framing, the study emphasizes transparent evaluation and a clear separation of roles. The tabular model captures the screening signal present in the questionnaire, while the graph organizes cases into a structure that supports querying, comparison, and interpretation. Together, these components provide a workflow in which prediction is complemented by contextual analysis rather than treated as a final decision.

## Results

The experiments were designed to distinguish rule-recovery performance from representation-based analysis while preserving the full NeuroCypher ASD pipeline. After deduplication and split-first evaluation, we examined six complementary components: (i) a tabular XGBoost reference baseline, (ii) a graph-only embedding model, (iii) a hybrid tabular-plus-graph model, (iv) graph ablations and weight-sensitivity analysis, (v) SHAP-based global and case-level interpretation of the graph classifier, and (vi) semantic querying over the knowledge graph.

### Reference Tabular, Graph-Only, and Hybrid Baselines

Three supervised baselines were evaluated with XGBoost under a common 70/30 split and 5-fold cross-validation protocol. The first was a reference tabular baseline using raw questionnaire and demographic variables. The second used only graph embeddings learned from the train-only knowledge graph. The third combined raw tabular variables with graph embeddings.

The tabular XGBoost reference baseline achieved very high holdout performance (ROC AUC = 0.997, F1 = 0.981, precision = 0.985, recall = 0.976, AP = 0.999). Its mean cross-validation ROC AUC on the training split was 0.997. However, because the published target label is deterministically derived from the questionnaire answers, this result should be interpreted as a strong reference baseline rather than evidence of independent diagnostic intelligence.

The hybrid raw-plus-graph model also achieved very high holdout performance (ROC AUC = 0.995, F1 = 0.969, precision = 0.958, recall = 0.981, AP = 0.998; CV ROC AUC = 0.995). This is consistent with the fact that the hybrid model inherits the highly informative questionnaire signal. By contrast, the graph-only baseline yielded substantially weaker holdout discrimination (ROC AUC = 0.585, F1 = 0.727, precision = 0.738, recall = 0.716, AP = 0.768), despite a somewhat higher training CV ROC AUC of 0.724. Thus, tuning improved graph-only cross-validation behavior but did not translate into strong holdout generalization.

Taken together, these findings suggest that the graph representation is not, by itself, a superior predictive substitute for the raw questionnaire on this dataset. Instead, its value is better understood in terms of structure, interpretability, and support for exploratory reasoning over case relationships. A summary of the holdout and cross-validation performance of the three supervised baselines is provided in Table 1.

**Table 1:** Holdout and cross-validation performance for the three main supervised baselines under the leakage-safer split-first protocol

Model	Holdout ROC AUC	F1	Precision	Recall	AP	CV ROC AUC
Raw tabular reference	0.997	0.981	0.985	0.976	0.999	0.997
Raw + graph	0.995	0.969	0.958	0.981	0.998	0.995
Graph only	0.585	0.727	0.738	0.716	0.768	0.724

### Graph Ablation Analysis

To examine how graph design choices affect performance, we compared several variants.

The full graph (with and without anomaly gating) achieved the best holdout performance (ROC AUC = 0.568, F1 = 0.824, precision = 0.730, recall = 0.947). Removing similarity edges reduced performance to ROC AUC = 0.524 (F1 = 0.813). The simplified graph gave ROC AUC = 0.483 (F1 = 0.830), while removing demographic information resulted in the lowest score (ROC AUC = 0.440, F1 = 0.811). Overall, graph structure affects the results, but the improvements are limited. Recall remains high, but discrimination ability (ROC AUC) is weak. This suggests that added context changes the learned representation, without clearly improving classification performance. Table 2 summarizes the effect of removing specific graph components on holdout performance.

**Table 2:** Ablation analysis of graph construction choices. Coverage and rejection-rate columns are retained because the anomaly-gated setting functions as a reject-option variant rather than a standard classifier

Graph variant	Holdout ROC AUC	F1	Precision	Recall	AP
Full graph pipeline	0.568	0.824	0.730	0.947	0.740
Full graph + anomaly gate	0.568	0.824	0.730	0.947	0.740
No similarity edges	0.524	0.813	0.710	0.952	0.755
Current graph pipeline	0.508	0.780	0.697	0.885	0.711
No behavior patterns	0.508	0.780	0.697	0.885	0.711
Simplified graph pipeline	0.484	0.830	0.710	1.000	0.715
No demographic context	0.440	0.812	0.707	0.952	0.679

### Weight Sensitivity

We further examined whether manually assigned edge weights materially affected graph-based performance. The best holdout configuration was a global +10% perturbation of the heuristic weights, with ROC AUC = 0.551, F1 = 0.825, precision = 0.709, recall = 0.986, and AP = 0.738. The heuristic baseline itself achieved ROC AUC = 0.532 and F1 = 0.821, whereas the uniform-weight configuration dropped to ROC AUC = 0.457 and F1 = 0.802. Other perturbations produced ROC AUC values between 0.500 and 0.539. The sensitivity analysis was therefore labeled *sensitive*, indicating that graph-based behavior depends on parameterization, even if absolute holdout discrimination remains limited.

An important observation is that several weight configurations produced high training CV ROC AUC values (approximately 0.91–0.95) while remaining close to chance on the holdout set. This divergence suggests that graph parameter tuning can easily overstate apparent performance if judged only on internal resampling, reinforcing the need for strict held-out evaluation. Table 3 summarizes the effect of alternative edge-weight configurations on graph-only performance.

**Table 3:** Sensitivity of graph-only performance to manual edge-weight configurations

Configuration	Holdout ROC AUC	F1	Precision	Recall	AP
All weights +10%	0.551	0.825	0.709	0.986	0.738
All weights -20%	0.539	0.759	0.720	0.803	0.748
Heuristic baseline	0.532	0.821	0.717	0.962	0.704
All weights -10%	0.521	0.808	0.708	0.942	0.755
Neutral similarity family	0.518	0.810	0.710	0.942	0.714
Neutral demographic risk weights	0.517	0.824	0.724	0.957	0.734
All weights +20%	0.500	0.780	0.720	0.851	0.695
Uniform weights	0.457	0.802	0.701	0.938	0.681

### Interpretability, SHAP Analysis, and Clinical Utility

SHAP explanations were generated for the same tuned graph-only XGBoost classifier reported in Table 1. Accordingly, the predictive context for this interpretability analysis is the graph-only baseline holdout performance already reported there (ROC AUC = 0.585, F1 = 0.727, precision = 0.738, recall = 0.716, and AP = 0.768). Although these scores do not support strong standalone prediction claims, the SHAP analysis remained useful for examining how the graph representation behaved.

At the global level, that is, across the holdout set as a whole, SHAP identified the embedding dimensions that contributed most strongly to the classifier’s predictions. The most influential dimensions were emb_29, emb_37, emb_87, emb_14, and emb_8. To improve interpretability, we computed approximate embedding-to-feature mappings based on correlation structure within the training data. These mappings suggested that the most influential embedding dimensions were associated with a mixture of demographic indicators and Q-Chat items, including ethnicity, family history of ASD, sex, and items such as A1, A3, A4, A5, A6, and A9. These associations are explicitly approximate and do not imply one-to-one semantic equivalence between embedding dimensions and clinical variables. The most influential embedding dimensions and their approximate bridges to original variables are summarized in Table 4.

**Table 4:** Top global SHAP dimensions for the graph classifier, with approximate bridges back to original questionnaire and demographic variables

Embedding dimension	Mean absolute SHAP	Approximate dominant mapped features
emb_29	0.269	Middle Eastern ethnicity, White European ethnicity, family ASD history
emb_37	0.206	A9, sex, A1
emb_87	0.196	A5, A3, A6
emb_14	0.194	Hispanic ethnicity, A1, A4
emb_8	0.157	family ASD history, A4, A6

Note: The table shows the most influential embedding dimensions based on mean absolute SHAP values. The mapped features indicate which original questionnaire or demographic variables are most strongly associated with each embedding dimension, based on correlation analysis. These associations are approximate and do not represent direct one-to-one mappings. A1–A10 correspond to the ten behavioral items of the Q-Chat-10 screening questionnaire, each recorded as a binary response (0/1)

At the local level, that is, for individual case predictions, SHAP identified the embedding coordinates that most strongly influenced a given decision. Across high-confidence positive holdout cases, influential local dimensions frequently included emb_29, emb_37, emb_87, emb_14, emb_51, and emb_78. An illustrative high-confidence positive case is shown in Table 5, where the prediction was driven primarily by emb_87, emb_78, emb_14, and emb_91. These case-level explanations support comparison across patients and inspection of model-specific patterns, functioning as a transparency layer over the graph representation.

**Table 5:** Illustrative local explanation for a high-confidence positive holdout case. The mapped original variables are approximate correlation-based bridges rather than exact semantic decompositions of the embedding space

Case ID	Embedding dimension	SHAP value	Approximate bridge to original variables
674	emb_87	0.869	A5, A3, A6
674	emb_78	0.300	Asian ethnicity, A6
674	emb_14	0.225	Hispanic ethnicity, A1, A4
674	emb_91	0.179	Black ethnicity, White European ethnicity, A9

Note: The table shows a local explanation for a single high-confidence positive case (Case ID 674). SHAP values indicate how each embedding dimension contributed to the prediction for this case, with positive values pushing the prediction toward ASD traits and negative values in the opposite direction. The mapped variables represent the original questionnaire or demographic features most strongly associated with each embedding dimension based on correlation analysis. These associations are approximate and do not correspond to direct or unique mappings. A1–A10 refer to the ten behavioral items of the Q-Chat-10 questionnaire, recorded as binary responses (0/1)

### Anomaly Detection and Decision Support

The anomaly-detection module is integrated into the deployed NeuroCypher ASD pipeline, where it serves as a supporting mechanism to identify atypical cases or data profiles deviating from the learned graph-embedding distribution. In the ablation study, an anomaly-gated evaluation variant was also tested. Although this variant did not improve holdout ROC AUC beyond the full graph pipeline, it preserved the same discrimination level while offering a reject-option design for decision support. This is clinically relevant because the practical utility of anomaly detection lies less in maximizing average predictive performance and more in highlighting cases that may require closer inspection, follow-up review, or data-quality verification.

From a clinical perspective, the findings indicate that high predictive accuracy in questionnaire-based ASD screening should not be conflated with richer diagnostic reasoning when the target variable is constructed from the same behavioral inputs. Under this interpretation, the principal contribution of NeuroCypher ASD lies in transforming tabular screening data into a structured and explorable graph representation that can support comparison of cases, inspection of local patterns, and transparent discussion of behavioral profiles in practice.

### Semantic Querying and Graph Exploration

The integrated natural-language interface provided direct access to the graph, enabling users to perform structured queries without requiring prior expertise in Cypher. In practice, this component enables clinicians and educators to query counts, subgroups, and case patterns directly through natural language, while the underlying system translates user intent into graph queries over behavioral and demographic nodes. Although the semantic interface was not re-evaluated as a predictive module, it remains central to the framework because it exposes the graph structure in a clinically accessible form. This supports the use of NeuroCypher ASD as an exploratory and explanatory environment rather than only as a predictive model.

To make the graph-based explanation layer more concrete, Table 6 presents an example derived from the Q-Chat-10 dataset and the same similarity logic used in the knowledge graph construction. For an index case, the graph can retrieve neighboring cases connected through highly overlapping questionnaire-response patterns and then expose the specific Q-Chat items shared across those cases.

**Table 6:** Illustrative graph-based case explanation showing an index case, retrieved similar cases, shared Q-Chat responses, and the resulting interpretation

Index case	Similar case	Similarity weight	Shared Q-Chat responses	Graph-based interpretation
619	530	1.000	A1–A10	Same questionnaire profile; sex, ethnicity, jaundice history, and family ASD history are also shared.
619	729	0.925	A1–A10	Same questionnaire profile with shared sex, jaundice history, and family ASD history.
619	90	0.930	A1, A2, A4–A10	Highly overlapping behavioral pattern with all four demographic fields also shared.

In this example, the graph explanation does not replace the model output. Instead, it makes the prediction easier to inspect by showing which cases are structurally close to the index case and which behavioral responses create that connection.

## Discussion

The results of this study show that NeuroCypher ASD is better understood as a decision-support system rather than a standalone predictive model. Its main contribution is that it connects a screening result with tools that help users understand the case, compare it with similar cases, and inspect patterns in the data. The framework brings together machine learning, a knowledge graph, and a natural-language interface in a form that can be used in practical settings such as schools. In this setup, prediction, interpretation, and data exploration are treated as distinct but connected steps.

The predictive results help clarify the role of each component in the system. The tabular model provided the strongest predictive performance, while the graph-based models added value by organizing cases in a way that could be explored and compared. This pattern is consistent with the structure of the dataset, where the target label is derived directly from the questionnaire responses. In this setting, machine learning primarily translates the questionnaire signal into probabilistic estimates, while the graph representation captures broader structural relationships between cases. This makes the components complementary within the same workflow: one summarizes the screening signal, and the other provides context for interpretation. For this reason, the knowledge graph should not be interpreted as a competing predictive model. Instead, its contribution lies in supporting comparison across cases, pattern inspection, and contextual interpretation. The evaluation of tabular, graph-only, and hybrid models was used to examine these roles explicitly, rather than to establish predictive superiority.

The natural-language interface strengthens this workflow by making structured data easier to use. This makes structured data more accessible to educators and therapists, who can explore patterns without relying on predefined reports. Prior work has shown that NLP tools can improve usability and engagement in similar contexts [13, 27]. In our case, the value of the interface lies in enabling simple questions to reveal structured insights from the data. Users can move directly from a screening result to case comparison, feature review, and simple exploratory questions.

Knowledge graphs are particularly useful because they organize the data around relationships rather than isolated entries. This allows users to move from isolated data points to relationships between cases. For example, similar behavioral profiles can be identified and compared directly, which is difficult to achieve with tabular views alone. This supports a clearer understanding of screening data, especially for non-technical users [11, 12]. In practice, this allows users to compare similar cases, inspect shared questionnaire responses, and explore demographic patterns in a structured way.

The anomaly detection module adds a further layer of support by drawing attention to profiles that differ from the broader pattern of the dataset. This is useful for highlighting profiles that may require closer review, especially when patterns are not immediately obvious. Previous work has shown the value of anomaly detection in identifying rare or ambiguous cases [8, 18]. Within NeuroCypher ASD, this feature helps users identify cases that may benefit from additional review.

Taken together, the system supports a workflow in which a prediction is first produced, and then examined in context. Users can explore similar cases, inspect shared features, and query the dataset directly. This shifts the focus from isolated predictions to interpretable decision support. The result is a more informative screening process in which the output can be examined, explained, and discussed.

From a practical perspective, this approach is well suited to school environments. Professionals can use the system to screen cases, explore patterns, and identify potential concerns without needing advanced technical skills. For teachers, therapists, and school support staff, this means that an initial screening result can be followed by a clearer view of why a case may need attention. For parents and caregivers, it can make the result easier to understand by showing patterns and similarities across cases rather than presenting only a single score. The goal is not to replace clinical assessment, but to support earlier and more informed referrals. This aligns with previous work emphasizing the importance of accessible tools in early ASD identification [1, 28].

A simple usage scenario illustrates the practical value of this workflow. A practitioner uploads or enters a new case and receives a prediction. They can then examine similar cases in the graph to assess whether the child’s profile matches a recognizable pattern in the dataset. For example, if the new case shows the same responses on items such as A1 (response to name), A5 (pretend play), and A9 (simple gestures) as several nearby cases in the graph, the prediction is not considered only as an isolated score; rather, the practitioner can see that it is consistent with a combination of behavioral responses that already appears across multiple similar cases. This can help the practitioner explain more clearly why the case may warrant closer attention. By contrast, if the prediction is positive but the case shows weak similarity to nearby cases in the graph, or if anomaly analysis flags it as unusual, the result can be interpreted more cautiously. In such situations, the system does not determine the decision, but it indicates that the case may benefit from closer review, additional observation, or another screening step before stronger conclusions are drawn. In this way, the system supports more transparent interpretation of predictions rather than automated decision-making.

Several limitations should be noted. First, the dataset is derived from a screening tool where the target label is computed from the same inputs. This limits the interpretation of predictive performance and does not reflect independent diagnostic capability. Second, the dataset is relatively small and lacks the diversity of real clinical data. External validation on real-world datasets is needed to assess generalizability. Third, graph-based prediction did not show strong performance in this setting. This suggests that graph representations are more suitable for structural analysis than for direct classification in this type of data. Fourth, the natural language interface performs well for simple queries but struggles with more complex reasoning tasks. Improving multi-step queries and aggregation remains an open challenge. Future work should therefore focus on larger and more varied datasets, stronger graph querying, and evaluation in real-world school settings to assess effects on understanding, communication, and referral decisions.

Overall, this work presents a system that connects prediction with interpretation and data exploration in a single workflow. By combining tabular machine learning with graph-based context, the framework supports more transparent and practical ASD screening in real-world settings.

## Data Availability

The dataset used in this study is publicly available at: https://www.kaggle.com/datasets/fabdelja/autism-screening-for-toddlers.

## Appendix A: The Q-Chat-10 Questionnaire and the Description of Demographic Variables

**Table 7:** Mapping of dataset variables to Q-Chat-10 questions

Variable	Corresponding Q-Chat-10 Question
A1	Does your child look at you when you call his/her name?
A2	How easy is it for you to get eye contact with your child?
A3	Does your child point to indicate that s/he wants something? (e.g., a toy out of reach)
A4	Does your child point to share interest with you? (e.g., at an interesting sight)
A5	Does your child pretend? (e.g., care for dolls, talk on a toy phone)
A6	Does your child follow where you’re looking?
A7	If someone in the family is visibly upset, does your child show signs of wanting to comfort them?
A8	Would you describe your child’s first words as:
A9	Does your child use simple gestures? (e.g., wave goodbye)
A10	Does your child stare at nothing with no apparent purpose?

Note: For items A1–A9, responses of *Sometimes*, *Rarely*, or *Never* are scored as 1; *Always* or *Usually* as 0. For A10, *Always*, *Usually*, or *Sometimes* are scored as 1. A total score above 3 suggests potential ASD traits

**Table 8:** Description of demographic variables

Variable	Description / Possible Values
Age_Mons	Child’s age in months (numerical value)
Sex	Biological sex of the child: *M* (Male), *F* (Female)
Ethnicity	Ethnic background: e.g., *White European, Hispanic, Black, Asian, Middle Eastern*
Jaundice	History of neonatal jaundice: *yes, no*
Family_mem_with_ASD	Family history of ASD: *yes, no*
Who_completed_the_test	Person completing the test: e.g., *Parent, Health Care Professional, Self, Others*

## Materials Availability

All materials used in this study are either publicly available or described in sufficient detail in the Methods section to allow replication.

## Code Availability

To promote research transparency and reproducibility, the complete implementation—including data preprocessing procedures, graph construction algorithms, embedding generation processes, model training pipelines, and evaluation protocols—has been made publicly available through a GitHub repository (https://github.com/GiorgosBouh/llm2cypher-asd-app). The repository features a comprehensive README file that thoroughly documents the codebase, detailing the specific function of each script as referenced in this paper, and provides the access link to a live, interactive instance of the NeuroCypher ASD application.
